# Dr. Langslow, in Reply

**Published:** 1802-03

**Authors:** R. Langslow

**Affiliations:** Halesworth


### *To the Editors of the Medical and Phyſical Journal.*

GENTLEMEN,

AMONGST the numerous Papers and Letters the preſent Controverſy upon Apoplexy has given birth to, none has excited my aſtoniſhment more than that which appeared in your laſt Number, with the ſignature of Dr. Girdleſtone annexed; as an Advertiſement, to nearly a ſimilar import, had been publiſhed in the Ipſwich Journal of the 26th of December laſt, accompanied by another from Mr. Crowfoot; in the latter of which I was charged with having deviated from the *original queſtion*; and in the former Dr. G. in addition to what he has now ſtated, appeared to ſupport the opinions of Mr. C. that Apoplexy is often a ſymptomatic Diſeaſe, meaning, as I ſuppoſe, with his friend Mr. C. that it is generally occaſioned by an oppreſſed or foul ſtomach, particularly as he immediately afterwards intimates, that the ſpecies of Apoplexy ariſing from extravaſation is a *ſuddenly fatal* diſeaſe, and can therefore rarely, if ever, come under the cognizance of the medical practitioner. I ſaw theſe Advertiſements in the Ipſwich Journal, on Sunday morning the 27th of December, 1801, and anſwered them the

*ſame* day, in ſuch terms that I flattered myſelf would convey conviction to the mind of every *unprejudiced* Reader; that Dr. G. was unneceſſarily captious, in objecting to the uſe of inverted commas to a paſſage where, if the Printer had made uſe of *Italics* inſtead of *ſuch* commas, no objection could poſſibly have been made. My Reader will, however, be better enabled to judge of my ſucceſs on this occaſion, by a peruſal of a Copy of the Letter I allude to, and which I truſt, from the eſtabliſhed impartiality and candour your Journal has acquired, will find as *ready* admiſſion into your *next* Number, as Dr. G's found in your *laſt*.

I am, &c.

*Haleſworth*, 8*th Feb.* 1802. R. LANGSLOW.

---

*A Copy of a Letter addreſſed to Dr.* GIRDLESTONE *and Mr.* CROWFOOT, *written upon the* 29*th of December*, 1801, *and inſerted in the Ipſwich Journal*, 2*d of January*, 1802.

The Letter being already before the Public, and Dr. Langſlow's Work on this Subject being expected in a few days, we only give his additional Obſervations, viz.

I am at a loſs to account for Dr. G's motive in tranſmitting to you a Paper for inſertion in your Journal, the *principal* contents of which had been anſwered previouſly to the date of it; and it is ſtill *more* extraordinary, that he neither alludes to it, or attempts the refutation of the Arguments there adduced; and as they have never ſince been noticed by Dr. G. I truſt the Public will naturally infer, that what I have there advanced is unanſwerable. Be this as it may, I ſhall not follow his example, but endeavour ſtill farther to explain and account for the *ſeeming* contradictions which that Letter of Dr. G's contains. Dr. G. at page 149, of your laſt Number, denies that Mr. Crowfoot's Pamphlet is the joint production of himſelf and Mr. C. In anſwer to which, I have to declare *poſitively*, that it was the general report in this neighbourhood *ſome weeks* before it appeared, that Dr. G. was not only a ſupporter of Mr. C's Doctrines upon Apoplexy, but that he was alſo aſſiſting in the Compoſition of the expected Work, and correcting and reviſing the ſheets to and from the Preſs; and there is no perſon who compares Mr. Crowfoot's firſt Work, "The Statement, &c." with the ſecond Publication, called, "The Obſervations, &c." but what will be inſtantly ſtruck with *the difference of diction and ſtile*, and muſt inſtantly exclaim, theſe *two* Publications poſſeſs the moſt ſatisfactory *internal evidence*, that they were *not* the production of the *ſame* Pen. But this is not all; all, the Friends of Mr. C. boafted, that Dr. L. would now meet with his match, or words to that effect, for that an eminent Phyfician had efpoufed the caufe of Mr. C. and was affifting in the Compofition of a Work which was totally to refute what had been publifhed upon Apoplexy by Dr. L. This was the triumphant language of Mr. C's. friends to thofe who happened to defend my fide of the queftion; and amongft others, I have to name the Rev. Mr. Bohun, of Bungay, Mr. C's brother-in-law, who, I was informed from *various* quarters, had declared publicly that Dr. Girdleftone was the redoubted Champion alluded to. And, on this occafion, I went to Bungay, with the intention of enquiring into the truth of this report, and applied to a perfon in that town of ftrict veracity, who told me inftantly, that he had not only heard it from others, but that Mr. B. had himfelf ftated to him that Dr. G. was correcting and revifing the Work, and that a fheet, fo corrected and revifed, had been fent to him, Mr. B. He farther ftated, that Mr. B. did not mention this fact in confidence, or enjoin fecrecy, and he therefore conceived himfelf at full liberty to anfwer my queftions without referve, or at the rifk of giving offence. After fuch clear proofs of the fact, no one, I truft, can condemn me for believing what I had heard, though I muft add, that if Dr. G. when he fo kindly undertook the tafk, did at the fame time exact fecrecy, or if Mr. C. and his friends promifed *not* to divulge it, then indeed it muft be admitted, they have acted ungratefully as well as treacheroufly, and on this account the Doctor has an undoubted claim upon the pity and compaffion of the public in this particular inftance.

The other quotations and anfwers alluded to in Dr. G's paper, I conceive to be completely explained in my letter to Dr. G. and Mr. C. inferted in the Ipfwich Journal and copied as above, I fhall, therefore, decline troubling your readers farther at prefent upon the fubject of Dr. G's paper, and proceed to anfwer Mr. Wilkinfon and Mr. Atkinfon, whofe papers on the fubject of Apoplexy appeared in your laft Journal, and fhall truft to your ufual impartiality and candour for their appearance in the *next* Number of your truly valuable Work.

---

### Dr. LANGSLOW, in Anfwer to Mr. WILKINSON, upon APOPLEXY.

When I firft began the perufal of this Paper, I confidered it as an attempt of its Author, to fupport the Doctrines of Mr. C. on Apoplexy; but when I arrived to near the bottom of page 141, I difcovered that Mr. W. had *forgot* which of the Difputants he *firft* meant to defend; for he there fays, " The ingenious Dr. Kirkland confidered that fort of Apoplexy to which Dr. L. alludes, to be a Difeafe, *fui generis.*" Now, unfortunately for Mr. W. it fo happens, that, that is the kind of Difeafe, *if fuch exifts*, which Mr. C. alludes to; for Dr. L. alludes *only* to Apoplexy arifing from *preffure upon the brain*, and he admits no other; nay, Mr. W. allows *the Nervous Affection* of Dr. Kirkland, and his converts Dr. G. and Mr. C. to be a Difeafe *fui generis*, *therefore*, I fhould conclude, *cannot* be Apoplexy; and as Mr. W. ftates that this *extraordinary* fpecies of Apoplexy, if Apoplexy it *muft* be, may originate in the thorax or abdomen, furely then, it might with *equal* propriety, and certainly with *greater fafety*, be claffed with St. Vitus's Dance, and of a nature *fui generis*; for by claffing it with the latter affection, it will ftill be fituated amongft Nervous Difeafes, and if erroneous, the error will not be productive of fuch dangerous confequences as might arife from claffing it with Apoplexy, otherwife a young and inexperienced Practitioner may, upon being called to a Cafe of *real* Apoplexy, hefitate to determine whether his patient is labouring under *real* or *nervous* Apoplexy; and fhould the latter idea ultimately prevail, he might, by neglecting to abftract blood, generally and topically, rifk the recovery of his patient. Now Mr. C. did not venture to defcend below the ftomach for the origin of Apoplexy, but Mr. W. does not hefitate to include the abdomen; and perhaps the next advocate for Mr. C. may be inclined to extend *the origin* of Apoplexy to the fphincter ani, or even the great toe! But to be ferious, Mr. W. next obferves that Mr. Chefhire had diffected many apoplectic patients, in whom he found no morbid appearance in the brain. Ought he not then to have inferred that thofe patients died from fome other difeafe with apoplectic fymptoms? And I am perfuaded that many perfons who have expired fuddenly, have been fuppofed to die of Apoplexy, when the heart or fome other vital organ has been the feat of difeafe. Nay, Mr. W. ftates, at page 142, " that in *all* thofe Cafes related by *his* and *my* worthy Preceptor, Dr. Denman, and called the Apoplexy of Pregnancy, there was *no* extravafation *upon the brain*, but that *the heart*, in *all* of them, was found *unufually* flaccid and without a fingle drop of blood in either auricles or ventricles." Is it not then more reafonable to infer, that thefe patients were carried off by fome morbid affection of the latter vifcus, than by Apoplexy, tho' their diffolution might have been fudden, and attended with apoplectic appearances? A Work I have now in the Prefs on this fubject, together with my profeffional avocations, preclude me attending Mr. W. through the whole of his very long and elaborate Paper; but but what I have at prefent omitted to notice, will probably be adverted to, and commented upon, at fome future opportunity.

*Halefworth, 7th Feb.* 1802.

---

### Dr. LANGSLOW, in Anfwer to Mr. ATKINSON, on APOPLEXY.

Dr. L. has great fatisfaction in acceding to the propriety, liberality and candour, which are apparent in *most* parts of Mr. A's paper; and notwithftanding he is at prefent much preffed for time, thinks it incumbent upon him to devote a few minutes to the examination of *those* points, in which Mr. A. at prefent feems to entertain opinions at *variance* with thofe of Dr. L. In page 152, Mr. A. fays, "That had there been extravafation in Mr. C's cafe, is it poffible it could be abforbed, though after bleeding, fo as to produce an immediate mitigation of every diftreffing fymptom?" Now, the mitigation was *not* immediate, neither were *all* the diftreffing fymptoms removed. The patient had complained the *whole* morning, of pain, oppreffion, vertigo, tenfion, and ftricture *through her head*, as fhe expreffed it, and which fymptoms gradually increafed till near dinner time, at which period fhe was obferved to make incorrect and inappropriate anfwers; this circumftance was particularly remarked by her brother-in-law, who thought from her *unusual* mode of anfwering him, that he had offended her; fhe had little appetite, and of courfe ate but a fmall quantity at dinner, though immediately afterwards fhe dropped down fenfelefs, and would have fell to the floor had fhe not been prevented. Her breathing became laborious and ftertorous, her jaw fixed, her pulfe full and rather flow, and fhe remained totally infenfible till after the arrival of Mr. Primrofe, who upon being queftioned by the hufband pronounced the cafe, *I think with great propriety, Apoplexy.* Mr. C. who was the family furgeon, was fent for from a diftance of feven miles, but in the interim Mr. P. bled her. The difeafe being of fo alarming a nature, it was determined to difpatch another meffenger eleven miles, to requeft my immediate attendance. Mr. C. however, having a fhorter diftance to travel, arrived before me, and, as Mr. P. affures me, approved *his opinion*, and what had been done; and then ordered the emetic. Now I learnt from Mr. P. that in about half an hour after the abftraction of blood from the arm, and about two hours from the attack, fhe *began to recover* her fenfes, fo as to anfwer queftions in *some degree* rationally, though ftill comatofe, and complaining of great pain, ftricture, and tenfion through the head, attended with fuch fenfations fations as fhe never before experienced, or will ever be erafed from her memory; her refpiration was ftill laborious and in fome meafure ftertorous, and thefe fymptoms remained, fometimes better, and at others worfe, till after the application of the leeches the following morning, though from the latter operation fhe received fpeedy and manifeft relief. Now, it will I think be admitted by Mr. A. that half an hour was fufficient time for the abforbents to remove *such* a portion of the extravafated lymph, as would admit of *a partial* reftoration of the fenforial energy; it furely cannot be requifite for the *whole* of the compreffing fluid to be reabforbed to effect *such* a purpofe. Allow me for the fake of argument to fuppofe, that in the cafe of this lady, exudation of lymph had begun to take place early in the morning, when the fymptoms of approaching Apoplexy commenced, and that the firft tea fpoonful *(or any given quantity)* produced the head-ach fhe at firft complained of, and that the caufe of exudation ftill remaining, a fecond fpoonful was extravafated; the pain, as *was the fact*, would be increafed, together with fymptoms of ftricture and oppreffion, till a third fpoonful caufed perhaps vertigo and a confufion of ideas; the fourth, an increafe of all thefe, with the addition of a tendency to infenfibility; and it is probable the fifth may produce the acme of difeafe, or fuch complete compreffion of the medullary fubftance of the brain, as totally to arreft the fenforial functions and bring the patient fenfelefs to the floor. Let us fuppofe this to be the period Mr. P. was fent for to this lady. Upon arriving he abftracts blood from the arm; is it unreafonable to fuppofe the effect of this operation diminifhed the determination to the brain, and by emptying the veffels, allowed the abforbents to act with increafed power, at the moment the exhalants defifted to exude an extra proportion of lymph? And if this is admitted, who will refufe to allow, that in half an hour they might have reabforbed fuch a quantity of fluid as would bring the patient back to the fame ftate fhe was in when the fourth fpoonful was exuded, as reprefented in the above fcale, namely, a tendency to infenfibility, &c. &c. but *with* an *ability* to fpeak or anfwer a queftion, as fhe was not deprived of this function till the fifth fpoonful was extravafated; therefore it muft be allowed reafonable to infer, that the moment the compreffing caufe was diminifhed by the abforption of the firft tea fpoonful, the effect of the fifth or laft exuded tea fpoonful would be obviated, and the patient reftored to the fame ftate of fenforial energy which exifted at the time the fourth fpoonful compreffed the brain; and indeed the patient remained under fuch effects as we ftated were produced by the fecond and third fpoonful exuded, till the leeches were applied the following day, after which, which, if we may judge from the ſymptoms, the reſidue of extravaſated lymph was rapidly removed. It therefore appears to me, that the grand ſource of the difference of opinion between Mr. A. and myſelf, has ariſen from the difficulty Mr. A. met with in accounting for the *ſpeedy* alleviation of *ſome of the ſymptoms* in this caſe, and his ſuppoſing that it was neceſſary that *the whole* of the extravaſated fluid muſt have been reabſorbed before even *the approach* to a mitigation of the ſymptoms could have been obſerved; and it is ſurely moſt probable to infer, that the laſt ſymptom ariſing from compreſſion of the brain will be the firſt to diſappear as the cauſe diminiſhes.

I formerly ſtated in my Explanation, No. 35 of the Medical Journal, that I did not preciſely accord with Dr. G. Fordyce in his opinion, that Apoplexy rarely occurs from any other ſource but effuſion of *red blood* upon the brain; neither do I think *exactly* with Dr. Cullen, that even expanſion, congeſtion, or fulneſs of the veſſels of the head, will produce *complete* Apoplexy. That a tendency to it, with many of the previous ſymptoms, ſuch as pain, ſtricture, tenſion, coma, and vertigo, may from ſuch a cauſe ariſe, but I much doubt that it will ever completely arreſt the ſenſorial energy, and occaſion the patient to fall ſenſeleſs to the floor. Be this however as it may, it will be ſatisfactory to mankind to know that theſe little hypothetical variations in opinion between Dr. Cullen, Dr. Fordyce, and myſelf, are of ſuch a nature as to require *no* practical difference in the mode of treatment, therefore, ſcarcely of ſufficient moment to diſpute *ſeriouſly* upon. But the Caſe is extremely different with the hypotheſis of Dr. Kirkland, Dr. Girdleſtone, and Mr. Crowfoot; it is of the utmoſt importance to determine upon the *real* exiſtence of nervous Apoplexy, ariſing *without preſſure upon the brain*, as the former aſſerts, and ſeems to be ſupported by Dr. G. and Mr. C. together with the utility of emetics in *any* Caſe of *real* Apoplexy, though in this laſt opinion I do not obſerve Mr. C. to be ſeconded by *any one* of his friends except Dr. G.; and this was the *original* ſource of diſpute between *him* and myſelf. Dr. Cullen, Dr. Fordyce, &c. agree with me in poinion, compreſſion of the brain is the general cauſe of Apoplexy; and I again call upon Dr. G. and Mr. C. to ſtate, *when* and *where* I diſcovered an inclination to relinquiſh the opinion I *firſt* advanced, that when this compreſſion was ſo complete as to bring the patient ſenſeleſs to the floor, it was *always* occaſioned by extravaſation of either blood or ſerum, and generally the latter. This was my firſt opinion, and I have not, as yet, expreſſed a deſire to abandon it. In the treatment of Apoplexy, whether the compreſſing cauſe is blood or ſerum, or turgeſcence of the veſſels of the head, the ſame remedies remedies will be indicated, namely, to abftract blood from the fyftem, both generally and topically, *in proportion* to the ftrength, conftitution, and former habits of the patient, in order as fpeedily as poffible to diminifh the determination to the head, and immediately aftterwards to empty the inteftinal canal by injections, and fuch laxatives, and fuch dofes as are not likely to excite the action of vomiting, but on no account whatever to adminifter an emetic, as fuch an operation muft determine the blood more ftrongly towards the head, and as powerfully impede its return from thence. The farther treatment of this Difeafe, together with my fentiments upon the dangerous doctrine of admitting the Nervous Apoplexy of Dr. Kirkland to exift, will be brought forward hereafter.

Mr. A. at page 153, ftates it to be probable, " that of ten cafes of Apoplexy which are *not fatal*, not above one or two is caufed by extravafation of blood upon the brain." Suppofing this to be the fact, it could only operate againft the theory of Dr. G. Fordyce; it would in no refpect affect *mine*, as in moft of thofe cafes which terminate happily, I have already ftated it as my opinion, that the caufe of the difeafe is exuded ferum. Nay, the cafe which Mr. A. himfelf has adduced at pages 153 and 154 of the laft Journal, is ftrongly illuftrative, and a powerful confirmation of *my* hypothefis. E. Trinder, (whofe cafe is fo accurately related by Mr. A.) there is little doubt, labored under *serous* Apoplexy; and we have ftrong caufe to believe that the ftimulating, joined with the evacuating, powers of the blifters, contributed to enable the abforbents to remove a portion of the exuded ferum, their ftimulation exciting the abforbing veffels at the fame time that their evacuating property emptied the blood veffels, and diminifhed the determination to the head; and thefe good effects were fo fpeedy, that we can only fuppofe that a *small* portion of exuded lymph, perhaps not a fifth or fixth part of the quantity extravafated, was taken up, *before* the good effects became apparent, by enabling her to fpeak rationally; but it required a *considerable time longer* for the compreffing fluid to be removed, fo as to diffipate *all* the remaining fymptoms. Is it poffible for any fact more ftrikingly to elucidate and confirm the doctrines I have been endeavoring to eftablifh, than the cafe here related? That the faculty of fpeech is commonly the laft which quits an apoplectic patient, and, upon *my* principle of reafoning, ought to be the *first* that returns, except where Apoplexy is fucceeded by Paralyfis, then indeed the refult may vary, as the organs of fpeech in that cafe may partake of the latter affection. Mr. A. ftates, that though E. Trinder recovered her fpeech in a *few hours* after the blifters were applied, which was as foon as we could reafonably expect pect they could produce *any* effect; yet that fome days elapfed before the fenforial energy was reftored to its priftine vigour, or, in other words, before the whole of the extravafated lymph which compreffed the brain was removed.

I cannot, however, take leave of Mr. A. without ftating my opinion, that he might have omitted the *concluding* portion of his, in many parts, well written and intelligent Paper, without injury to his reafoning powers, or his liberality as a fcholar and a gentleman. His reflections upon Univerfity honours, when *regularly* and *fairly* obtained, befpeaks, I am inclined to think, jealoufy and envy; and what he means by a long etcetera of Fellowfhips, Titles, and Diploma Splendours infatuating the multitude, I am at a lofs to comprehend, unlefs it is his intention to join Philo-Medicus in his laudable, well-timed, ingenious, and pointed fatire upon fuch diplomas as are conveyed from the North by mail-coachmen and carriers. It is not, however, unlikely, I may have incurred the difpleafure of Mr. A. by ftating, that it may be deemed by *some*, derogatory for a Phyfician to contend with an Apothecary: It is poffible Mr. A. might have miftaken my *meaning* in *this* fentence, by fuppofing I intended to reflect upon the *whole* body; which is certainly erroneous; for when he thinks me writing *contemptuously* to Mr. C. it is *merely* the refult of *his* conduct towards me. When a phyfician is called to a patient, as in the prefent inftance, and happens to prefer the application of leeches to the ufe of an emetic, will it not be deemed by Mr. A. himfelf, derogatory for the phyfician to contend with the apothecary upon the propriety of the one, or the impropriety of the other, whilft *under* the roof of the patient? It is furely, at fuch a time, the duty of the apothecary to comply with the directions of the phyfician without a murmur, or an attempt at remonftrance. The moment a phyfician appears in the fick chamber, he *alone* becomes refponfible; and if fo, ought he not to poffefs the *sole* direction of the mode of procuring relief to the patient? However, if the apothecary upon this, or any other occafion, difcovers *marked* proofs of defective judgment and fkill, he may at a future time, if he poffeffes talents, ability, and education fufficient for the purpofe, detect and expofe, with propriety, *such* deficiences in the phyfician; and it will no longer be derogatory for the phyfician to contend with the apothecary in clearing himfelf, if in his power, from fuch an attack: Nay, was he to decline it, or fhrink from it, he would ftand felf-convicted before the tribunal of the public; and which muft have been my own fate, had I refufed to anfwer Mr. C's *first* publication. It was affuredly infra dignitatem to agree with Mr. C. in the houfe of the patient; but the moment he attacked tacked me *publickly*, it was incumbent upon me to defend myfelf, and fuch an act was no longer derogatory. We have, however, but too much reafon to believe, that Mr. C. was piqued that the hufband of the lady fhould deem it neceffary to require aid, which *he conceived* to be no way fuperior *to his own*, particularly when the emetic ordered by *himself* was forbidden. His conduct on this occafion, and the numerous mifreprefentations exhibited in his firft publication, have perhaps rendered my ftile of writing more fevere and contemptuous than I fhould have made ufe of to another apothecary, who might have conducted himfelf with greater propriety; as I can without hefitation affirm, no one entertains a greater refpect for *many*, *very many*, individuals in the Chirurgical and Pharmaceutical Profeffions than myfelf: I am acquainted with great numbers, whofe acquirements would do honour to the character and title of phyfician; and to fuch I fhall *ever* think it my duty to behave with that deference and refpect they fo juftly merit. But when *others* conduct themfelves improperly, and affert that no perfon has a right to confult a phyfician, without *first* obtaining the confent and approbation of his apothecary, it is time for the phyfician to defend his own rights, and treat fuch apothecaries with the contempt fuch declarations merit.—A work I fome time fince publifhed, has placed this conduct of certain gentlemen in a confpicuous point of view, and, probably, contributed to occafion the prefent Controverfy, which confirms the old adage, That good frequently originates from evil. And I cannot drop my pen, without regretting the anxiety expreffed by Dr. Girdleftone to relinquifh this important controverfy prematurely, and before the utility of Emetics in this difeafe, and the exiftence of the Nervous Apoplexy of Dr. Kirkland, are eftablifhed, or *for ever* configned to oblivion. Surely, fuch anxiety indicates fears and apprehenfions for the refult of the ultimate decifion; and which, I hope, will not be relinquifhed till the defirable object it embraces is obtained.

Dr. L. has now to ftate, that the Lady whofe cafe has been the fubject of the prefent interefting Controverfy, has experienced a *second* attack of the fame difeafe; the progrefs of it, however, was intercepted before it arrived at its acme by the *timely* application of leeches to the temples and a proper laxative, without any other remedy being indicated. She left her chamber with a *similar* head-ach, ftricture, vertigo, &c. and which *progressively increased* till fhe began to give inappropriate anfwers, at *which period* the effects of the abftraction of blood from the head became evident, and kept the difeafe ftationary, till the laxative operated; and fhe was perfectly free from from difeafe the fucceeding morning. Have we not reafon then to conclude, that if fhe had been bled generally and topically, in the *firft* attack, a fhort time before fhe became fenfelefs, that fuch an event would have been prevented, and the difeafe have been as fpeedily and as fafely removed as in the *latter* inftance?

