# Errata

**Published:** 1802-04

**Authors:** 


### ERRATA.

Vol. VII. p. 275, l. 2, from the bottom, for *forgot* read *forgotten.*

p. 281, l. 2, from the bottom, for *agree* read *argue.*

